# Sodium Channel Gene Mutations in Children with GEFS+ and Dravet Syndrome: A Cross Sectional Study

**Published:** 2013

**Authors:** Seyed Hassan TONEKABONI, Ahmad EBRAHIMI, Mohammad Kazem BAKHSHANDEH BALI, Seyedeh Mohadeseh TAHERI OTAGHSARA, Massoud HOUSHMAND, Mohammad Mahdi NASEHI, Mohammad Mahdi TAGHDIRI, Mehdi MOGHADDASI

**Affiliations:** 1 Professor of Pediatric Neurology, Pediatric Neurology Research Center, Shahid Beheshi University of Medical Science, Tehran, Iran; 2 PhD of Medical Molecular Genetic, Research Institute of Endocrine Sciences, Shahid Beheshi University of Medical Science, Tehran, Iran; 3 Fellow of Pediatric Neurology, Pediatric Neurology Research Center, Shahid Beheshti University of Medical Sciences, Tehran, Iran; 4 General Physician,Tehran University of Medical Sciences,Brain and Spinal Injury Research Center, Neuroscience Institute, (TUMS), Tehran, Iran; 5 PhD of Medical Clinical Genetic, National Institute of Genetic Engineering and Biotechnology, Tehran, Iran; 6 Assistant Professor of Pediatrics, Pediatric Neurology Research Center, Shahid Beheshti University of Medical Sciences (SBMU),Tehran, Iran; 7 Associate Professor of Pediatric Neurology, Shahid Beheshti University of Medical Science, Tehran, Iran; 8 Neurologist, Department of Neurology, Rasool-e-Akram Hospital, Tehran University of Medical Sciences, Tehran, Iran

**Keywords:** Dravet syndrome, GEFS+, SCN1A mutations

## Abstract

**Objective:**

Dravet syndrome or severe myoclonic epilepsy of infancy (SMEI) is a baleful epileptic encephalopathy that begins in the first year of life. This syndrome specified by febrile seizures followed by intractable epilepsy, disturbed psychomotor development, and ataxia. Clinical similarities between Dravet syndrome and generalized epilepsy with febrile seizure plus (GEFS+) includes occurrence of febrile seizures and joint molecular genetic etiology. Shared features of these two diseases support the idea that these two disorders represent a severity spectrum of the same illness. Nowadays, more than 60 heterozygous pattern SCN1A mutations, which many are de novo mutations, have been detected in Dravet syndrome.

**Materials & Methods:**

From May 2008 to August 2012, 35 patients who referred to Pediatric Neurology Clinic of Mofid Children Hospital in Tehran were enrolled in this study. Entrance criterion of this study was having equal or more than four criteria for Dravet syndrome. We compared clinical features and genetic findings of the patients diagnosed as Dravet syndrome or GEFS+.

**Results:**

35 patients (15 girls and 20 boys) underwent genetic testing. Mean age of them was 7.7 years (a range of 13 months to 15 years). Three criteria that were best evident in SCN1A mutation positive patients are as follows: “Normal development before the onset of seizures, onset of seizure before age of one year, and psychomotor retardation after onset of seizures.

Our genetic testing showed that 1 of 3 (33.3%) patients with clinical Dravet syndrome and 3 of 20 (15%) patients that diagnosed as GEFS+, had SCN1A mutation.

**Conclusion:**

In this study, normal development before seizure onset, seizures beginning before age of one year and psychomotor retardation after age of two years are the most significant criteria in SCN1A mutation positive patients.

## Introduction

Charlotte Dravet was the first scientist who described severs myoclonic epilepsy in infancy (SMEI) in 1978 at Marseille (1). The disorder has a prevalence of about 1/400,000 and is responsible for approximately 7% of severe epilepsies with seizure onset before age 3 years (2). Criteria for diagnosis of Dravet syndrome obtained from international league against epilepsy (ILAE) are as follows: 1- normal development before seizure, 2- seizure onset before one year of age, 3- multiple seizure type, 4-family history of epilepsy or febrile convulsion, 5- abnormal EEG findings, 6- psychomotor retardation after age 2 years, 7- ataxia and pyramidal signs, 8- anticonvulsant resistance, and 9-exacerbation of seizure with fever (3). 

GEFS+ or generalized epilepsy with febrile seizure plus is a familial epileptic syndrome inherited as an autosomal- dominant characteristic that even in a genotypically identical family has a great phenotypic heterogeneity pattern. The most prevalent clinical manifestation of GEFS+ is febrile seizure that may appear after the age range of classical febrile convulsion (3 months to 6 years) and can be accompanied by multiple types (myoclonic, atonic or partial) of seizure (4,5).

Dravet syndrome and GEFS+ are two characteristics of one spectrum, while Dravet syndrome presents more severe clinical manifestations than GEFS+ (6). These two epilepsy syndromes are most commonly related to mutation in sodium channel gene famili subunits (SCN), including GEFS+ on the minor end and Dravet syndrome on the major end of the spectrum (7).

In patients clinically recognized as Dravet syndrome, mutations in SCN1A gene are detected in 33-100% of cases. The sodium channel α1-subunit encoding protein acts as a neuronal voltage-gated sodium channel α1-subunit encoding gene. Voltage-gated sodium channel α1-subunit is dominantly presented in the central nervous system (8). This study was designed to provide guidance for physicians, pediatrics, and pediatric neurologists to diagnose the patients with Dravet syndrome or GEFS+ and to provide appropriate indications for mutation analysis of the SCN1A gene. To this purpose, we compared clinical features of patients diagnosed as Dravet syndrome and GEFS+.

## Materials & Methods

From May 2008 to August 2012, 35 patients who referred to Pediatric Neurology Clinic of Mofid Children Hospital in Tehran were enrolled in this study. Entrance criterion of this study was having equal or greater than four characteristics of Dravet syndrome criteria obtained from Commission on Classification and Terminology of the International League Against Epilepsy (ILAE).

Any form of epilepsy with abnormal EEG findings and positive family history of febrile and nonfebrile seizures with exacerbation of seizure with fever were the inclusion criterias for GEFS+ .Any form of epilepsy with abnormal EEG findings and positive family history of febrile and nonfebrile seizures with exacerbation of seizure with fever were the inclusion criterias for GEFS+.

All patients were aware of this study and informed consent was signed by parents before evaluations. 

The primary evaluation included history taking, physical examination (general and neurologic), pedigree charting, electroencephalography (EEG), laboratory testing (biochemical and metabolic assay) and magnetic resonance imaging (MRI).

Three milliliters of whole blood was collected from the patients and their parents. Then, DNA was extracted from peripheral blood leukocytes using salting-out procedure described by miller et al. (9) and was qualified by Biophotometer, Eppendorf Spectrophotometer 000576, Eppendorf Company, Hamburg, Germany. All coding regions of SCN1A were amplified using intronic primers and the products were sequenced directly by ABI Sequence Analyzer 3130. 

All negative samples for sequence variations and point mutations were screened by multiplex ligationdependent probe amplification (MLPA) for detection of SCN1A deletions, Insertions or large gross rearrangements (10-12).

After genetic study, we have followed all the patients with known mutations according to their clinical course and evolutionary status until now. We analyzed clinical pictures and genetic features by Fisher’s exact test using SPSS software.

## Results

Thirty five patients (15 girls and 20 boys) underwent genetic testing. Mean age of them was 7.7 years (a range of 13 months to 15 years). Four features of ILAE criteria that were more pronounced in these patients were as follows: 1- multiple seizure types (33 of 35, 94.2%), 2- positive family history of epilepsy or febrile seizure (30 of 35, 85%), 3- resistance to multiple anticonvulsants (27 of 35, 77.1%), 4- exacerbation of seizures with hyperthermia (27 of 35, 77.1%). Table one presents description of all patients on the basis of ILAE criteria. Based on clinical evaluations, these patients were classified into five categories as follows: atypical febrile convulsion (5.7%), generalized epilepsy febrile seizure plus (85.7%), and Dravet syndrome (8.6%). After genetic study, SCN1A mutation was found in four patients, three belonging to GEFS+ spectrum and one from Dravet group.

The average age of seizure onset was 14.5 months (a range of 45 days to 4 years), while these four mutation positive patients manifested their first onset of seizure before age of one year (an average age of 8.4 months). 

All mutation positive patients and 38.7% of the mutation negatives had normal development before seizure onset. According to psychomotor retardation after 2 years of age, all of the mutation positives and 37.1% of the others had these criteria. 

Concerning these three characteristics (seizure onset before age 1 year, normal development before seizure onset, and psychomotor retardation after age 2 years), there were significant differences between mutation positive and mutation negative patients (p<0.05).

Our genetic testing showed that 1 of 3 (33.3%) patients with clinically Dravet syndrome and 3 of 20 (15%) patients diagnosed as GEFS+, had SCN1A mutation. The further confirmed molecular genetic screening of child clinically categorized as Dravet showed a heterozygous missense in exon 2 as p.S103G, which causes a serine to glycine substitution in N-terminus of SCN1A gene and results in Dravet syndrome (13). 

Molecular analysis of the first patient with diagnosis of GEFS+ showed a novel unreported heterozygous sequence variation in codon 412, D1/S6 transmembrane domain of SCN1A protein, causing a phenylalanine to isoleucine substitution. Novel missense in codon 1274 resulting in a tyrosine to asparagine substitution with heterozygous novel sequence variation occurring in exon19, D3/S2 transmembrane domain of SCN1A gene was detected in the second patient diagnosed as GEFS+. Molecular study of the third patient with features of GEFS+ revealed a missense as p.R101Z that resulted in arginine to glutamine substitution occurring in the N-terminus conserved domain of SCN1A gene (13).

**Table 1:** Clinical Characteristics of Patients

**No**	**sex**	**Age**	**Epilepsy type**	**Normal develop Before seizure**	**Seizure** **Begin** **Before 1 year**	**Multiple Seizure type**	**Family history Of epilepsy/ FC**	**Abnormal EEG findings**	**Psychomotor Retardation After age 2 years**	**Ataxia and** **Pyramidal signs**	**Anticonvulsant resistance**	**Exacerbate of seizure with ferer**
1	F	13m	GEFS	+	+	+	-	+	-	-	+	-
2	M	3.5y	GEFS	+	-	+	+	+	-	-	+	+
3	M	10 y	GEFS	+	-	-	+	+	-	-	+	-
4	F	10 y	GEFS	-	-	-	-	+	+	-	+	+
5	M	8 y	GEFS	-	-	-	+	+	+	-	-	+
6	M	8.5y	GEFS	+	-	-	+	+	-	-	+	+
7	F	6y	GEFS	+	-	-	+	+	-	-	+	+
8*	M	15y	GEFS	+	+	-	+	+	+	-	+	+
9	F	9y	GEFS	+	+	+	+	+	+	-	+	+
10	F	4.5y	GEFS	-	+	-	+	+	-	-	-	+
11	M	10.5y	GEFS	-	-	-	+	+	-	-	+	+
12	M	9y	Draret	+	+	-	-	+	+	-	+	+
13	F	12y	GEFS	-	-	+	+	+	-	-	+	-
14	F	5y	GEFS	-	+	-	+	+	-	-	+	-
15	F	7y	Dravet	+	+	+	+	+	+	-	+	+
16	M	11y	GEFS	-	+	-	+	+	-	-	-	+
17*	M	10y	Dravet	+	+	+	+	+	+	+	+	+
18	F	6y	GEFS	-	-	+	+	+	-	-	+	+
19	E	7.5y	GEFS	+	+	-	+	+	-	-	-	+
20	F	9y	FC	-	+	+	+	-	-	-	-	+
21	M	8 y	FC	-	-	+	+	+	-	-	-	+
22	E	5 y	GEFS	-	+	+	+	+	+	-	-	+
23	M	9y	GEFS	+	+	+	+	+	+	-	+	+
24*	M	5.5y	GEFS	-	-	+	+	+	+	-	+	+
25	M	6y	GEFS	-	-	-	+	+	-	-	+	+
26	M	8y	S.E	-	+	-	-	+	+	-	+	-
27	F	10y	GEFS	-	-	-	+	+	+	-	-	+
28	M	9y	GEFS	+	+	-	+	+	-	-	+	+
29	M	15y	GEFS	+	+	-	+	+	-	-	+	+
30	M	6.5y	GEFS	-	-	+	+	+	-	-	+	-
31	M	8y	GEFS	-	-	-	+	+	+	-	+	+
32	M	7y	GEFS	+	-	-	+	+	-	-	+	+
33*	F	9y	GEFS	+	+	+	+	+	+	+	+	+
34	F	9.5y	GEFS	-	-	+	+	+	-	-	+	-
35	M	13y	GEFS	-	-	+	+	+	-	-	+	-

GEFS+= generalized epilepsy with febrile seizure plus,*= SCN1A mutation positive patients

## Discussion

In this study, the three criteria that were best evident in SCN1A mutation positive patients were as follows: normal development before the onset of seizures, onset of seizure before age of one year, and psychomotor retardation after onset of seizures. These findings of the current study are consistent with those of Fountain- Capal et al. who found three criteria, which best differentiated between mutation positive and mutation negative children. The three criteria comprised exacerbation with hyperthermia, normal development before seizure onset and ataxia, pyramidal signs or interictal myoclonus representation (14). Hattori et al. declared an age of febrile seizure onset less than or equal to 7 months, a total seizure number more than or equal to 5, and seizures lasting more than 10 minutes were the main risk factors for Dravet syndrome (15). 

Another important finding was that mutations in SCN1A were evident in 33.3% of our patients clinically diagnosed as Dravet syndrome. This frequency is in agreement with Nabbout et al.’s findings which showed a frequency of 35% (16). The SCN1A mutation frequency in Fountain-Capal et al.’s studied patients was 23% (16 of 69 children) that is lower than that of ours (14). 15% of patients who were clinically diagnosed as GEFS+ had SCANIA mutation. A Japanese groups detected mutation in SCN1A in 77-82% of patients with severe myoclonic epilepsy of infancy (Dravet) (17). However, approximately 35% of Dravet cases and 5-10% of GEFS+ cases had SCN1A mutation in Australian, French, Canadian, and Italian studies (18). In our study, SCAN1A mutation was detected in 28.5% of children with febrile seizure onset before one years of age. In a study by Hattori et al., 6 of the 50 (12%) patients who had febrile seizure before their first year’s birthday, had SCN1A mutations (15). 

Fujiwara et al. reported clinical features anticipating a worse developmental outcome in patients with Dravet syndrome included status epilepticus, interictal abnormalities within the first year of life, electroencephalography and motor disorder (19). 

The average age of seizure onset in our mutation positive patients was 8.4 months and prior study by Dravet et al. and Engel. J. Jr et al. emphasized that the first seizure of these patients occurred at 5-8 months of age, while in a study of Fountain-Capal et al the seizure onset of 69 patients with Dravet syndrome was 2-16 months (14,20). In the two mutation positive patients, reported molecular genetic findings were able to corroborate clinical manifestations. Contrary to the previously published investigation, there is some equivocation in penetrance and clinical presentation. This hesitancy raised by the third patient which diagnosed as GEFS+ as well. This patient was clinically diagnosed as GEFS+, while molecular study suggests a Dravet syndrome phenotype (21). These two novel heterozygous suite variations containing p.F412 I and p.Y1274N in D3/S2 domain diagnosed respectively in the first and second patients with GEFS+, may be more different from known mutations. In some studies, missense substitutions in D1/S6 domains in the first patient result in epileptic manifestations particularly Dravet syndrome, however, one study suggested that this domain mutation leaded to cryptogenic generalized epilepsy (22). Mutations reported in D3/ S2 domain detected in the second patient, usually cause both Dravet syndrome and GEFS+; however, clinical manifestations is more compatible with GEFS+ (23). 

**In conclusion,** in this study, normal development before seizure onset, seizures beginning before age one year, and psychomotor retardation after age two years are the most significant criteria in SCN1A mutation positive patients.
